# Neoadjuvant chemoimmunotherapy in locally advanced gastric or gastroesophageal junction adenocarcinoma

**DOI:** 10.3389/fonc.2024.1342162

**Published:** 2024-04-15

**Authors:** Xiao Liu, Baozhen Ma, Lingdi Zhao

**Affiliations:** ^1^ Radiotherapy Department, The Affiliated Cancer Hospital of Zhengzhou University & Henan Cancer Hospital, Zhengzhou, China; ^2^ Immunotherapy Department, The Affiliated Cancer Hospital of Zhengzhou University & Henan Cancer Hospital, Zhengzhou, China

**Keywords:** neoadjuvant, chemoimmunotherapy, gastric or gastroesophageal, locally, adenocarcinoma

## Abstract

Patients suffering from locally advanced gastric or gastroesophageal junction adenocarcinoma often face a high postoperative recurrence rate. Despite aggressive treatment, less than 50% survive beyond five years. Ongoing clinical studies are exploring ways to prolong patient survival, revealing that perioperative chemotherapy can extend both the period of recurrence-free survival and overall survival for this group of patients. Currently, combining chemotherapy and immune checkpoint inhibitors has become a critical treatment approach for advanced gastric or gastroesophageal junction adenocarcinoma. However, the effectiveness of this approach in locally advanced patients remains unverified. This article delves into the latest research concerning the use of perioperative chemotherapy coupled with immune checkpoint inhibitors in locally advanced gastric or gastroesophageal junction adenocarcinoma treatment, and highlights prospective challenges and discusses how to best identify patients who may benefit from combined chemotherapy and immune checkpoint inhibitor therapy.

## 1 Introduction

Gastric cancer is a global health concern, resulting in over a million new cases and approximately 769,000 deaths in 2020. It’s the fifth most common and fourth deadliest cancer worldwide (1). Locally advanced gastric or gastroesophageal junction adenocarcinoma (LAG/GEJA) are marked by tumors extending beyond the muscle layer or involving lymph node metastases, but without distant spread (2–4). Treatment typically involves perioperative chemotherapy, radiochemotherapy, and surgical intervention (4). These treatments aim to manage the disease from various perspectives, including reducing the cancer stage to increase surgical removal possibility, eliminating microscopic cancer cell clusters, enhancing complete tumor resection chances, and lowering recurrence risk (5, 6). Even so, the prognosis of patients with LAG/GEJA is still poor, with most patients relapsing within three years and a disappointing five-year survival rate of less than 50% (7–9). Recurrences are often due to residual tumors or microscopic metastases undetected during surgery (10). Therefore, there is an urgent need to improve clinical outcomes for patients with locally advanced gastric cancer (7–9).

Chemotherapy not only exerts cytotoxic effects on tumor cells but also reshapes the tumor microenvironment, exhibiting a synergistic effect when combined with immune checkpoint inhibitors (ICIs) (11). ICIs have significantly revolutionized the treatment paradigm for numerous advanced cancers, emerging as a pivotal component in disease management. This transformation is particularly pronounced in the context of advanced gastric or gastroesophageal junction (G/GEJ) adenocarcinoma. The incorporation of anti-PD-1 antibodies with chemotherapy, such as sintilimab and nivolumab, has demonstrated substantial advantages (12, 13). Meanwhile, ICIs have also improved pathological complete response (pCR) rates and disease-free survival (DFS) in melanoma, non-small cell lung cancer (NSCLC), and triple-negative breast cancer (14–17). Although there has been progress in the integration of neoadjuvant chemotherapy and ICIs for gastric cancer, as of now, no randomized controlled phase III clinical trials have reported positive outcomes. This review delineates the recent developments in chemoimmunotherapy for the perioperative management of LAG/GEJA. Additionally, it addresses the existing challenges in this field.

## 2 Clinical study of neoadjuvant chemoimmunotherapy in LAG/GEJA

### 2.1 Neoadjuvant chemotherapy in LAG/GEJA

The MAGIC study represents a significant milestone in the therapeutic approach to LAG/GEJA. This study examined patients with LAG/GEJA, as well as lower esophageal adenocarcinoma. Participants were stratified into two cohorts: one undergoing solely surgical intervention and another receiving perioperative treatment. The perioperative cohort received three cycles of ECF chemotherapy both preoperatively and postoperatively, and exhibited a marked improvement in 5-year overall survival rates compared to those in the surgery-only group (36% vs. 23%) (8). This finding was supported by the FFCD9703 study (9). Moreover, the FLOT-AIO study revealed that FLOT outperformed ECF as a preoperative treatment, resulting in superior pCR rates and OS (7, 18). Concurrently, the RESOLVE and PRODIGY studies, undertaken in Asia, demonstrated that perioperative chemotherapy markedly prolongs DFS in patients with LAG/GEJA (19, 20). Collectively, these investigations underscore the critical importance of perioperative chemotherapy in the management of LAG/GEJA. However, whether perioperative chemotherapy is the most effective treatment strategy remains under scrutiny. As our comprehension of the molecular intricacies of gastric cancer deepens, the spectrum of treatment is expanding to encompass targeted therapies and immunotherapies. These emerging treatment modalities are currently being explored and validated, especially for early-stage gastric cancer due to the poor 5-year survival rate (7).

### 2.2 Neoadjuvant targeted therapy in LAG/GEJA

Roughly 22% of patients with G/GEJ adenocarcinoma exhibit over-expression of Her2 (21), a condition that accelerates tumor progression via pathways such as PI3K/Akt/mTOR and MAPK (22). This overexpression, particularly prevalent in gastric cancer, correlates with more aggressive disease progression and inferior prognosis (23, 24). The landmark ToGA study represented a pivotal advancement in this realm, being the first to demonstrate that combining trastuzumab with chemotherapy significantly extends survival in advanced G/GEJ adenocarcinoma patients with Her2 gene amplification. This discovery heralded a new frontier in targeted therapy for advanced gastric cancer (21).

Building on this, the PETRARCA study delved into the perioperative clinical efficacy of integrating anti-Her2 dual-target treatment with FLOT chemotherapy, compared to FLOT alone, in patients with cT2-4 and/or N+ G/GEJ adenocarcinoma and HER2 overexpression. The results were promising, indicating a substantial improvement in the pCR rate when trastuzumab and pertuzumab were combined with FLOT chemotherapy (35% vs 12%, p = 0.02). Additionally, the incidence of pathological lymph node negativity was higher (68% vs 39%) (25). However, the trastuzumab/pertuzumab cohort encountered an increase in adverse events, notably severe cases of diarrhea and leukopenia (25). Despite these advancements, the negative outcomes of the JACOB trial in Her2 overexpressing advanced G/GEJ adenocarcinoma led to the premature discontinuation of the PETRARCA study (26). Currently, the Phase II INNOVATION study is in progress, aiming to assess the effectiveness of trastuzumab in conjunction with perioperative FLOT chemotherapy in treating resectable G/GEJ adenocarcinoma patients (27). The study, anticipated to be finalized by 2028, primarily aims to delve deeper into the effectiveness of trastuzumab in combination with chemotherapy for this specific patient group.

The role of trastuzumab in augmenting Her2 internalization and cross-presentation by dendritic cells has been established, leading to the activation of T cell responses targeting Her-2 (28). Additionally, trastuzumab exerts extra antitumor activity by impacting immune system, notably by promoting lymphocyte infiltration into tumors (29). Consequently, the hypothesized synergy between anti-Her2 targeted therapy and immunotherapy appears highly plausible (30). A perfect demonstration of this synergy was shown in the Keynote811 study, where patients with advanced Her2 over-expression G/GEJ adenocarcinoma were involved. Participants in this study were divided into two groups; one received pembrolizumab treatment while the other got a placebo, both groups were treated alongside trastuzumab and chemotherapy. The group that received pembrolizumab had a significantly higher objective response rate (ORR) of 74.4% vs 51.9%, and a complete response (CR) rate of 11.3% vs 3.1% (31). These outcomes suggest that the triple combination of chemotherapy, anti-Her2 therapy, and ICIs therapy may provoke a more potent tumor response. This integrative therapeutic strategy is becoming increasingly significant, particularly in the spheres of neoadjuvant and transformational treatments.

Preliminary data from a Phase II clinical trial shows promising results when using a combination of camrelizumab, trastuzumab, and chemotherapy as neoadjuvant therapy for Her-2 over-expressing G/GEJ adenocarcinoma. In this trial, a notable pCR rate of 31.3% was achieved in 16 patients who underwent D2 radical surgery (32). However, these findings should be approached with caution due to the limited number of participants and the short duration of follow-up. These constraints highlight the need for additional studies, encompassing a larger group of participants and longer observation periods, to validate these preliminary results robustly.

### 2.3 Neoadjuvant chemoimmunotherapy in LAG/GEJA

Chemoimmunotherapy has shown impressive efficiency as the first-line treatment for advanced G/GEJ cancer, particularly in patients with a PD-L1 combined positive score (CPS) of 5 or higher (12, 13). This synergistic approach, leveraging both anti-PD-1 antibodies and chemotherapy, has significantly improved ORR and OS (12, 13). These findings suggest that G/GEJ cancer, notably responsive to anti-PD-1 antibody treatment, may be particularly amenable to immunotherapy. Consequently, it is hypothesized that immunotherapy could potentially improve survival outcomes in patients with LAG/GEJA. This hypothesis is supported by the GERCOR NEONIPIGA study, which is conducted in LAG/GEJA patients with mismatch repair deficiency (dMMR) or high microsatellite instability (MSI-H). In this study, participants received four cycles of neoadjuvant nivolumab and ipilimumab, followed by surgical intervention. Post-surgical treatment involved nine cycles of adjuvant nivolumab. The primary objective was pCR. The study encompassed 32 patients, out of whom 29 successfully underwent surgery, all achieving R0 resection. Impressively, 17 patients reached pCR status (T0N0). Furthermore, after a median follow-up period of 14.9 months, no recurrences were observed (33). In parallel, the INFINITY study reported similar results, 9/15 patients with dMMR/MSI-H LAG/GEJA achieved pCR (34).

Contrasting with previous research, recent evidence indicates that patients with MSI-H LAG/GEJA experience inferior survival outcomes when undergoing perioperative chemotherapy compared to surgery alone. This is evidenced by a Hazard Ratio (HR) of 2.22, falling within a 95% confidence interval (CI) of 1.02 to 4.85, and a P value of 0.04 (35). Following this revelation, the CLASSIC study’s subgroup analysis, focusing on Asian participants, revealed that adjuvant capecitabine and oxaliplatin after D2 gastrectomy did not confer survival benefits for MSI-H patients. The 5-year DFS rates showed no difference with a P value of 0.931 (36). Further elucidation came from a comprehensive meta-analysis that included four neoadjuvant/adjuvant chemotherapy trials: MAGIC, CLASSIC, ARTIST, and ITACA-S. This analysis demonstrated that only non-MSI-H patients benefited from combining chemotherapy with surgery, compared to surgery alone. This was highlighted by a 5-year overall survival (OS) rate of 62% versus 53%, corresponding to an HR of 0.75, within a 95% CI of 0.60 to 0.94 (37). These findings compellingly suggest that neoadjuvant/adjuvant chemotherapy might not be the most effective treatment approach for patients with MSI-H LAG/GEJA. However, the GERCOR NEONIPIGA and INFINITY studies have reported significant benefits of perioperative immunotherapy, potentially establishing new treatment paradigms for patients with MSI-H LAG/GEJA (33, 34). Despite these promising outcomes, it is imperative to conduct more thorough clinical research to ascertain whether perioperative immunotherapy, either as a standalone treatment or in conjunction with neoadjuvant chemotherapy, can yield enhanced benefits for this specific patient group. This need for additional research is underscored by a retrospective study that documented a series of cases involving LAG/GEJA patients with MSI-H (38). In these cases, patients received a combination of chemotherapy and ICIs, resulting in favorable pathological responses. This observation suggests potential benefits but also highlights the necessity for more comprehensive and controlled studies to validate these findings and guide treatment strategies.

NCT0291816 is a phase 2, single-arm clinical trial aimed at evaluating the efficacy of a novel treatment regimen for patients with LAG/GEJA. Participants received three cycles of capecitabine and oxaliplatin, combined with pembrolizumab, followed by an additional cycle of pembrolizumab only prior to surgery. Postoperatively, patients continued pembrolizumab for up to one year. Of the 34 patients enrolled, seven achieved pCR (39). Meanwhile, the DANTE study, a multicenter phase II trial, investigated the clinical efficacy of perioperative chemoimmunotherapy compared to perioperative chemotherapy in patients with resectable LAG/GEJA, enrolling 295 patients. Early results showed comparable R0 resection rates. However, the chemoimmunotherapy group demonstrated a pCR rate of 24% and an MPR rate of 48%, compared to 15% and 39%, respectively, in the chemotherapy cohort. Further subgroup analysis revealed enhanced pCR and MPR rates among patients with PD-L1 CPS≥10 and MSI-H status (40). In another study identified as NCT04250948 explored the efficacy of combining toripalimab with SOX/XELOX in patients with LAG/GEJA, engaging 108 participants. Results indicated a significant increase in tumor regression grades 0/1 (TRG0/1) from 20.4% to 44.4%, and an elevation in the pCR rate from 9.3% to 24.1% (41). These preliminary results are promising and suggest potential advancements in treatment strategies. Further support comes from additional small-scale phase II clinical trials utilizing camrelizumab with FOLFOX and sintilimab with XELOX, which have reported primary outcomes in terms of pCR and MPR (42, 43). This body of research collectively underscores the potential of integrated treatment approaches, combining chemotherapy with immunotherapeutic agents, to improve outcomes for patients with LAG/GEJA. Regarding safety, a meta-analysis revealed that neoadjuvant chemoimmunotherapy, when compared to neoadjuvant chemotherapy alone, did not elevate the incidence of G3-4 treatment-related adverse events (TRAEs) or surgical complications in LAG/GEJA (44). In another real-world study, findings suggested that the addition of tislelizumab to neoadjuvant chemotherapy, as opposed to neoadjuvant chemotherapy alone, exhibited no significant differences in terms of surgical duration, number of resected lymph nodes, postoperative hospital stay, and 30-day mortality (45).

The Keynote-585 trial was designed to assess the efficacy and safety of combining pembrolizumab with perioperative chemotherapy in patients with LAG/GEJA. The primary endpoints were event-free survival (EFS) and pCR. The latest findings from this study were presented at the 2023 ESMO Conference. The data revealed a striking increase in the pCR rate when pembrolizumab was added to chemotherapy, compared to chemotherapy alone (12.9% vs 2.0%, p<0.00001). Additionally, the pembrolizumab group demonstrated an extended EFS compared to the control group (44.4 vs 25.3 months, p=0.0198). however, this difference did not reach the predetermined threshold for statistical significance, indicating that the observed variation in EFS was not statistically significant (46). In parallel, the MATTERHORN study, a global, multicenter, randomized, controlled Phase III clinical trial, investigates the efficacy and safety of the neoadjuvant therapy incorporating either durvalumab or a placebo, combined with the FLOT regimen in patients with resectable G/GEJ adenocarcinoma, followed by adjuvant therapy with durvalumab or placebo. The primary endpoint of this study is EFS. Preliminary results, also shared at the 2023 ESMO conference, showed a notable increase in the pCR rate for patients with early and locally advanced G/GEJ adenocarcinoma. This improvement was achieved through the combination of durvalumab with the FLOT regimen, yielding a pCR rate of 19% compared to just 7% in the absence of durvalumab (p<0.00001) (47). The EFS results from the MATTERHORN study are still pending. Once released, these findings will be crucial in assessing the potential benefits of integrating ICIs with standard perioperative chemotherapy in reducing recurrence rates and improving survival outcomes for patients with LAG/GEJA. **Table 1** presents clinical studies with preliminary results on neoadjuvant chemoimmunotherapy and in LAG/GEJA, and **Table 2** details the ongoing randomized studies of neoajuvant chemoimmunotherapy in LAG/GEJA.

**Table 1:** Selected neoadjuvant clinical studies administering chemoimmunotherapy for patients with LAG/GEJC.

Study	Year	Study type	Study name/ No. Register	Sample size	Clinical stage	Treatment	Primary endpoint	pCR rate
Guo, et al. (48)	2022	phase 2	ChiCTR2000030414	30	cT3-4NanyM0	Sintilimab + XELOX	pCR	33.30%
Jiang, et al. (49)	2022	phase 2	NCT04065282	36	cT3-4NanyM0	Sintilimab +XELOX	pCR	19.40%
Yin, et al. (50)	2022	phase 2	NCT04890392	32	cT3-T4aN+M0	Tislelizumab+SOX	MPR	25%
Tang, et al. (51)	2022	phase 2	Neo-PLANET	36	cT3-4aNany M0	Camrelizumab+ concurrent CRT	pCR	44.40%
Li, et al. (52)	2023	phase 2	NCT03878472	25	T4a/bN+M0	Camrelizumab + apatinib + SOX	pCR	15.80%
André, et al. (33)	2023	phase 2	GERCOR NEONIPIGA	32	cT2-4NxM0	Nivolumab + Ipilimumab	pCR	58.60%
Janjigian, et al. (47)	2023	phase 3	MATTERHORN	948	>T2N0-3M0/T0-4N1-3M0	FLOT ± Durvalumab	pCR, EFS	19.00%
Shitara, et al. (46)	2023	phase 3	Keynote 585	804	cT3-4NanyM0	FP/XP ± Pembrolizumab	pCR, EFS	12.90%
Wei, et al. (53)	2023	phase 2	ChiCTR1900024428	34	cT3-4bN+M0	Sintilimab + concurrent CRT	pCR	38.20%
Verschoor, et al. (54)	2024	phase 2	PANDA	21	cT3-4aNanyM0	Atezolizumab +DCF	Safety	45%
Yuan, et al. (55)	2024	phase 2	NEOSUMMIT-01	54	cT3-4aN+M0	Toripalimab + SOX/XELOX	pCR	22.20%

pCR, pathological complete response; MPR, major pathological response; CRT, chemoradiotherapy; XELOX, capecitabine plus oxaliplatin; SOX, tegafur plus oxaliplatin; FLOT, fluorouracil, docetaxel, and oxaliplatin; FP, fluorouracil plus cisplatin; XP, capecitabine pluls cisplatin; DCF, docetaxel, oxaliplatin, and capecitabine; EFS, event-free survival.

**Table 2:** Selected ongoing randomized studies of neoajuvant chemoimmunotherapy in LAG/GEJA.

Register number	Study type	Study name	Sample size	Clinical stage	Treatment	Primary endpoint
NCT04139135	phase 3	HLX10-006-GCneo	642	cT3N+M0	XELOX ± HLX10	EFS
ISRCTN15837212	phase 2/3	DANTE	556	≥cT2 and/or N+M0	FLOT ± atezolizumab	DFS
NCT04744649	phase 2	NICE	110	cT3-4aNxM0/cT2N+M0	XELOX/SOX ± toripalimab	MPR
NCT05101616	phase 2	Camrelizumab-GC-neoadjuvant	100	T3-4aN1-3M0	Nanopaclitaxel+SOX± Camrelizumab	MPR
NCT05699655	phase 2	TAOS-3B-Trial-2	130	Borrmann IV/Large Borrmann III Type/Bulky N +	SOX ± apatinib and tislelizumab	pCR
NCT04592913	phase 3	D910GC00001	958	≥Stage II (resectable)	FLOT ± durvalumab	EFS
NCT06155383	phase 2	RC48-C022	90	cT3-4aN+M0	XELOX/SOX± toripalimab ± RC48	pCR
NCT04195828	phase 2	Arise-FJ-G005	53	cT2-4aNanyM0	nab-paclitaxel +S1± Camrelizumab and Apatinib	MPR
NCT04661150	phase 2	ML42058	41	cT3-4bNanyM0	Trastuzumab+XELOX ± atezolizumab	pCR
NCT05161572	phase 2	NeoRacing	152	cT3-4aN+M0/cT4bNanyM0	SOX+sintilimab ± RT	pCR
NCT05149807	phase 2/3	SHR-1701-III-308	896	cT2-T3/N+M0	SOX ± SHR1701	pCR, EFS

XELOX, capecitabine plus oxaliplatin; SOX, tegafur plus oxaliplatin; FLOT, fluorouracil, docetaxel, and oxaliplatin; EFS, event-free survival; DFS, disease-free survival; MPR, major pathological response; pCR, pathological complete response.

## 3 Limitations of pCR as primary endpoint

Clinical trials exploring the effectiveness of neoadjuvant chemoimmunotherapy in LAG/GEJA are currently underway, usually with pCR as the primary endpoint. pCR is an important benchmark in neoadjuvant therapy, as studies have shown its association with improved long-term survival outcomes such as DFS and OS (56–61). However, it’s important to acknowledge that trials using pCR as the primary endpoint have certain limitations.

### 3.1 pCR may be unsuitable for all types of tumors in neoadjuvant therapy

pCR may not be universally applicable across all types of neoadjuvant therapies for certain tumors. While a strong correlation between pCR and improved long-term survival is evident in early-stage breast cancer and LAG/GEJA cases, a specific study on operable breast cancer suggested that pCR may not reliably predict long-term outcomes (62). Additionally, a recent meta-analysis found only a weak relationship between pCR and both EFS and OS in Her2 over-expression breast cancer cases (61). Further research is needed to understand the relationship between pCR and EFS/OS in LAG/GEJA cases with Her2 over-expression during perioperative therapy. Furthermore, the Keynote 585 study achieved one of its primary endpoints (pCR), but did not result in a significant improvement in EFS (46). This highlights the need for a more nuanced understanding of pCR’s role in different LAG/GEJA subtypes and treatment scenarios.

### 3.2 Tumor heterogeneity

The concept of tumor heterogeneity is a fundamental principle in oncology. Tumors often display significant variation, particularly in terms of their molecular and genetic characteristics. This heterogeneity is not only observed among different patients, but also within different regions of the same in a single patient. During treatment, it is common to observe varying responses from different parts of the tumor, with some areas showing growth while others regress. This variability may explain why a specific treatment can led to the completely elimination of certain tumor cells, resulting in a pCR, while other areas remain metabolically active. A partial response to treatment like this may contribute to a small percentage of patients experiencing relapse after achieving pCR through neoadjuvant therapy (63). This highlights the complexity of tumor treatment and underscores the need for personalized treatment plans that consider the intratumor differences within each tumor.

### 3.3 The incomplete measure of pCR in efficacy assessment

In certain scenarios, the advantages of neoadjuvant therapy go beyond achieving a pCR. Even when pCR isn’t fully achieved, patients may still derive notable benefits. These include the reduction of tumor size, consequently facilitating more manageable and shorter surgical procedures. Additionally, these benefits encompass a decrease in surgical complications and an extended duration of freedom from relapse. This holds particular relevance in cases of esophageal adenocarcinoma, especially in patients with pathologically lymph node-negative status (64). It underscores that lymph node regression following neoadjuvant chemotherapy serves as a robust in prognostic indicator. Moreover, retrospective analyses from the MAGIC study have identified lymph node negativity as an independent predictor of prolonged survival (65). This emphasizes that pCR isn’t the sole metric of assessing therapeutic success.

### 3.4 The diagnostic challenges of pCR

Despite the collaborative efforts of pathologists to standardize and precisely assess a pCR, the diagnosis process remains challenging. This challenge primarily stems from the subjective nature of the pathological diagnosis, introducing variability in result consistency. Critical factors, such as a thorough histological examination of the tumor bed and drainage lymph nodes, play a vital role in achieving an accurate pCR diagnosis. Nevertheless, ongoing debates persist regarding the optimal number of tumor bed sections and lymph nodes required for a conclusive assessment.

### 3.5 The risk of undertreatment in early pCR

The early achievement of pCR during treatment introduces unique challenges. Patients and physicians may make decisions that lead to under-treatment, influenced by the apparent success observed early in the course of therapy. Prematurely halting of treatment may, in turn, lead to relapse in specific patients. These challenges underscore the importance of vigilant monitoring and potential extension of treatment protocols, even when early signs of pCR are evident.

## 4 Optimizing neoadjuvant chemoimmunotherapy in LAG/GEJA

The concurrent administration of chemotherapy and ICIs has demonstrated significant effectiveness in treating various advanced and certain early-stage tumors (12–15, 31, 66–69). Currently, the standard approach involves administering these treatments simultaneously. However, to optimize the efficacy of this combination therapy, especially when integrating chemotherapy with ICIs, several crucial considerations need careful attention.

### 4.1 Selecting an appropriate chemotherapy regimen

The choice of chemotherapy regimen should be meticulously made, taking into account the specific type of tumor, its molecular characteristics, and the overall health status of the patient. In Europe, the FLOT regime, comprising three drugs, is commonly recommended for neoadjuvant chemotherapy in G/GEJ adenocarcinoma. This recommendation is largely influenced by the favorable outcomes of the FLOT-AIO study (7). In East Asia, a perioperative approach involving a two-drug regimen—either SOX or DOS—tends to be the standard protocol (19, 20). Younger and healthier patients capable of tolerating intensive treatment may consider a triple-drug regimen. Conversely, a less intensive two-drug combination such as SOX or DOS might be more apt for older patients or those in weaker health conditions.

### 4.2 Establish the optimal sequence and timing

Integrating chemotherapy with ICIs necessitates careful consideration of the sequence and timing of administration. Chemotherapy-induced tumor cell death releases tumor antigens, priming lymphocytes to identify and eliminate tumor cells presenting these antigens. Concurrently, ICIs amplify the proliferation and activation of these lymphocytes (70), rendering them more receptive to the effects of chemotherapy. Thus, the order of administration becomes strategically significant. The groundbreaking TONIC trial investigated the sequence of chemotherapy and ICI by assigning patients with triple-negative breast cancer to different pre-treatments, including low-dose radiotherapy, cisplatin, cyclophosphamide, doxorubicin, or a control observation period, before receiving nivolumab. The cohort treated with doxorubicin demonstrated the highest ORR (71). Additionally, a retrospective analysis of advanced lung cancer patients who received chemotherapy combined with ICIs showed improvement in progression-free survival (PFS) and OS. This improvement was observed when ICIs were administered 3-5 days after chemotherapy (72). These findings suggest that specific chemotherapeutic agents, coupled with the administration sequencing of chemotherapy and ICIs, can create a more inflammatory tumor microenvironment, potentially enhancing the response to immunotherapy.

Nivolumab, when administered as a monotherapy, activates anti-tumor T cells in the peripheral blood, with this specific T cell subgroup reaching its peak within one to two weeks after the initial dose. This surge is subsequently followed by a gradual decline (73). To alleviate the potential toxicity of second-cycle chemotherapy drugs on these activated T lymphocytes, extending the intervals between chemotherapy cycles would prove beneficial. Providing a longer period for the T cells to function may decrease the likelihood of adverse events, potentially leading to enhanced clinical outcomes.

### 4.3 The number of cycles in neoadjuvant therapy

It is pivotal to determine the appropriate number of neoadjuvant chemoimmunotherapy cycles for patients with LAG/GEJA. These individuals often suffer from reduced food intake, digestion, and absorption due to the distinctive response of their digestive tract to chemotherapy. These challenges can have adverse effects on their nutrition, leading to an overall decline in health. Such health deterioration may potentially compromise their ability to endure subsequent surgery and impede wound healing. Therefore, it is advisable to conduct routine assessments every 2-3 cycles during neoadjuvant therapy. These assessments serve as a basis for deciding whether to continue with the initially planned number of therapy cycles or make modifications. The decision is guided by the outcomes of these imaging assessments, aiming to maintain the patient’s overall well-being throughout the course of therapy.

### 4.4 Chemotherapy dosage

Chemotherapy drugs elicit an immune response by promoting the immunogenic death of tumor cells and creating an environment conducive to activated cytotoxic T lymphocytes through the elimination of Treg cells (74, 75). Given these advantages, it’s not recommended to decrease the dose of chemotherapy drugs in each cycle. Instead, a patient’s height and weight should significantly influence the drug dosage. In a clinical study on chimeric antigen receptor T (CART) cell therapy for relapsed/refractory non-Hodgkin lymphoma, patients who received pre-treatment with both fludarabine and cyclophosphamide before the CART cell transfusion exhibited superior results compared to those pre-treated with only cyclophosphamide. They had higher overall response and complete response rates. Additionally, the maximum tolerated dose of fludarabine and cyclophosphamide during pre-treatment led to tumor reduction in most patients with minimal severe toxicity (76). Laboratory data also revealed that patients pre-treated with both drugs had significantly higher numbers of CART cells in their peripheral blood than those pre-treated with just cyclophosphamide. This implies that lymphocyte-depleting chemotherapy before active cell infusion may enhance the elimination of immunosuppressive cells, thereby creating a conducive environment for the infused cells to exert their effects (76). However, it remains unclear if similar outcomes occur when chemotherapy is combined with ICIs. Nonetheless, in theory, comparable results might be anticipated in patients sensitive to ICIs therapy.

## 5 Challenges in neoadjuvant chemoimmunotherapy for LAG/GEJA

In the realm of LAG/GEJA, neoadjuvant chemoimmunotherapy presents a promising approach. It offers potential benefits like reducing tumor size, facilitating tumor downstaging, improving surgical results, and eliminating residual microscopic tumors. Despite demonstrating efficacy in a considerable number of cases, several challenges still warrant attention.

### 5.1 Selection patients for neoadjuvant chemoimmunotherapy

Determining the optimal candidates for neoadjuvant chemoimmunotherapy in patients with LAG/GEJA presents a significant clinical challenge. This difficulty largely stems from the absence of definitive predictive biomarkers to gauge the efficacy of such therapy in these patients. Without these biomarkers, it becomes challenging to identify those who would benefit most substantially. However, it’s noteworthy that studies focusing on first-line treatments for advanced gastric cancer have revealed considerable advantages of chemoimmunotherapy, especially in patients exhibiting a PD-L1 CPS of 5 or higher (12, 13). Consequently, there’s a growing recommendation to consider chemoimmunotherapy for patients with a PD-L1 CPS of 5 or more. In a phase II clinical trial at our center, which involved a limited number of participants, an impressive pCR rate of 48% (12 out of 25) was observed in LAG/GEJA patients with a PD-L1 CPS ≥ 1 (77). This suggests that neoadjuvant chemoimmunotherapy could potentially offer significant pCR benefits for this patient group, although this needs validation in a larger cohort. Additionally, findings from Keynote 811 indicate that combining chemoimmunotherapy with anti-Her2 therapy might yield pCR benefits for patients with Her2 overexpression (31). Our own unpublished retrospective data supports this, showing a notable pCR rate of 37.9% (11 out of 29) in LAG/GEJA patients treated with neoadjuvant chemoimmunotherapy in conjunction with anti-Her2 therapy. Moreover, dual immune neoadjuvant therapy has demonstrated substantial pCR benefits for LAG/GEJA patients with MSI-H (33, 34). Yet, comprehensive data on the efficacy of neoadjuvant chemoimmunotherapy in MSI-H patients remains scarce. Besides PD-L1 CPS, Her2 overexpression, and tumor mutation burden, multi-omics analysis also revealed several biomarkers predictive of pathological response, including RREB1 and SSPO mutations, immune-related signatures, and a peripheral T cell expansion score (52). In clinical practice, it is crucial to identify biomarkers such as PD-L1, Her2, MSI status, and tumor mutation burden in any accessible specimens. These markers are instrumental in guiding the choice of neoadjuvant therapy. Although these indicators provide valuable insights, ongoing research is essential to uncover additional biomarkers and enhance our understanding of the most effective therapeutic approaches for LAG/GEJA patients.

### 5.2 Adverse events

Adverse events associated with neoadjuvant chemoimmunotherapy can significantly impact a patient’s overall health and their ability to tolerate surgery. This form of treatment involves chemotherapeutic agents that can reduce patients’ resistance and increase the risk of secondary infections due to myelosuppression. Additionally, these agents may lead to gastrointestinal complications, potentially resulting in malnutrition. Furthermore, complications arising from the use of ICIs can also affect a patient’s surgical readiness. These complications might include effects on cardiopulmonary function, diminished adrenal cortex activity, and hypothyroidism. When these issues are combined, they can substantially reduce a patient’s capacity to withstand surgical procedures. Moreover, the adverse effects of neoadjuvant therapy may induce functional irregularities in other organs, which could emerge during the patient’s post-treatment period. This could potentially offset the survival advantages gained from the improved pCR and MPR rates that neoadjuvant therapy aims to achieve. Therefore, it is imperative to closely monitor patient tolerance and adverse events throughout the course of the therapy. Equally important is the need to promptly administer appropriate treatments as and when they become necessary. This careful management is essential to optimize patient outcomes and ensure the best possible balance between treatment efficacy and quality of life.

### 5.3 Efficacy assessment

The current assessment of clinical efficacy in neoadjuvant therapy is based on RECIST criteria, which were originally formulated for evaluating the efficacy in advanced solid tumors. In the context of neoadjuvant chemoimmunotherapy, accurately determining whether an increase in tumor size observed on imaging is a true progression or a pseudo-progression presents a significant challenge. This uncertainty can lead to the premature discontinuation of neoadjuvant therapy, prompting either an immediate surgical intervention or a shift to combined radiotherapy within the neoadjuvant treatment framework.

In terms of MPR, the residual tumor undergoes pathological changes, including fibrosis, lymphocyte infiltration, and the formation of tertiary lymphoid structures that resemble lymph node follicles (70). This transformation may cause an apparent enlargement of the tumor lesion on imaging scans. However, radiotherapy, though effective in destroying infiltrating lymphocytes, may inadvertently impair the activated anti-tumor immune response. Currently, clinical studies predominantly focus on pCR as the primary endpoint for neoadjuvant chemoimmunotherapy in patients with LAG/GEJA. However, the effectiveness of pCR as a metric has its limitations, and it remains uncertain whether a higher pCR rate directly correlates with improved EFS. Studies like Keynote585 and MATTERHORN have indicated that combining neoadjuvant chemotherapy with ICIs may increase pCR rates (46, 47). However, Keynote585 did not meet its predetermined EFS goals, failing to achieve one of its primary endpoints. Furthermore, the data on OS is still preliminary, necessitating additional research to confirm whether neoadjuvant chemoimmunotherapy indeed offers survival advantages for patients with LAG/GEJA.

### 5.4 Adjuvant therapy for patients achieving pCR

The efficacy of administering additional therapy for patients who achieve a pCR following preoperative chemoimmunotherapy remains an area yet to be definitively determined. In the era of preoperative chemotherapy, only a very small percentage of patients with LAG/GEJA achieved pCR. Historically, this particular group of patients often did not receive significant attention. According to established treatment protocols, all patients were subjected to additional therapy postoperatively (7, 19, 20). However, two patients with deficient mismatch repair (dMMR) experienced a recurrence of the disease during postoperative follow-ups, despite achieving a pCR after receiving SOX plus camrelizumab as neoadjuvant therapy (63). It is important to highlight that patients with dMMR typically have a relatively poor prognosis. Therefore, even in cases where a pCR is attained, the need for continued postoperative adjuvant therapy remains a critical consideration. In the current landscape of neoadjuvant chemoimmunotherapy, which is marked by a significant increase in pCR rates (for instance, a 19% pCR rate in the MATTERHORN study) (47), it is essential to conduct further research to determine whether postoperative adjuvant therapy can offer additional survival benefits to this distinct subset of patients.

### 5.5 Subsequent therapeutic strategies for patients in clinical CR by neoadjuvant chemoimmunotherapy

Patients undergoing radical gastrectomy for LAG/GEJA often face the necessity of removing their entire stomach or a substantial part of it. This surgery leads to not only nutritional challenges but also frequent occurrences of upper abdominal pain, bloating, nausea, vomiting, diarrhea, and dumping syndrome. Such complications significantly deteriorate their quality of life. In particular, after surgery, patients may experience severe esophageal reflux due to the loss of lower esophageal sphincter function, further impacting their well-being. Insights from research on MSI-H colorectal cancer suggest that patients achieving a clinical CR following neoadjuvant anti-PD-1 antibody therapy may be candidates for observation, postponing or even avoiding the need for immediate surgery and the attendant complications like colostomy (78, 79). This approach has shown promise in improving the overall quality of life for these patients. However, there is a lack of similar research concerning LAG/GEJA patients who achieve a cCR. The potential for these patients to be similarly managed through observation, thereby possibly delaying or avoiding gastrectomy and its associated life-altering effects, remains an uncharted area. Consequently, further research is imperative to explore these possibilities and improve treatment strategies for this specific patient subgroup.

## 6 Prospection

The amalgamation of chemoimmunotherapy is fundamentally reshaping the landscape of G/GEJ cancer treatment. Currently, the treatment approach, albeit somewhat conservative, predominantly involves adding ICIs into standard chemotherapy, and then comparing this combination to a placebo-plus-chemotherapy regimen, However, this method, while operationally straightforward, does not fully exploit the potential synergistic therapeutic benefits. There is a pressing need to optimize the dosage, frequency, and sequence of drugs in this combined therapy to maximize treatment efficacy. A deeper understanding of how chemotherapy agents affect the immune system is also crucial. In the treatment process, it’s imperative to explore biomarkers that could predict the response to treatment. These include PD-L1 CPS, Her2, MSI/dMMR, claudin18.2, VEGFR, NTRK, FGFR, c-MET, and EBV. Further investigation is warranted to determine whether ongoing adjuvant therapy contributes to improved survival in patients achieving pCR. Additionally, the role of surgery in the context of neoadjuvant chemoimmunotherapy requires reevaluation. The necessity and timing of surgery for patients who achieve a clinical complete remission is a critical question that remains unanswered, underlining the need for more in-depth research in this area.

## Author contributions

XL: Writing – original draft, Writing – review & editing. BM: Writing – original draft. LZ: Supervision, Writing – review & editing.
